# Supplementary material for: Winner–loser plant trait replacements in human-modified tropical forests
Source: Nat Ecol Evol. 2024 Dec 10;9(2):282–95. doi: 10.1038/s41559-024-02592-5 (PMC11807837; doi:10.1038/s41559-024-02592-5)
Supplement: Supplementary file 1 — Reporting Summary [file 41559_2024_2592_MOESM1_ESM.pdf]

nature portfolio

Corresponding author(s): Bruno X. Pinho & Jos Barlow

Last updated by author(s): Oct 21, 2024

Reporting Summary

Nature Portfolio wishes to improve the reproducibility of the work that we publish. This form provides structure for consistency and transparency in reporting. For further information on Nature Portfolio policies, see our Editorial Policies and the Editorial Policy Checklist.

Statistics

For all statistical analyses, confirm that the following items are present in the figure legend, table legend, main text, or Methods section.

n/a	Confirmed	
☐	☒	The exact sample size (*n*) for each experimental group/condition, given as a discrete number and unit of measurement
☐	☒	A statement on whether measurements were taken from distinct samples or whether the same sample was measured repeatedly
☐	☒	The statistical test(s) used AND whether they are one- or two-sided<br>*Only common tests should be described solely by name; describe more complex techniques in the Methods section.*
☐	☒	A description of all covariates tested
☐	☒	A description of any assumptions or corrections, such as tests of normality and adjustment for multiple comparisons
☐	☒	A full description of the statistical parameters including central tendency (e.g. means) or other basic estimates (e.g. regression coefficient) AND variation (e.g. standard deviation) or associated estimates of uncertainty (e.g. confidence intervals)
☐	☒	For null hypothesis testing, the test statistic (e.g. *F*, *t*, *r*) with confidence intervals, effect sizes, degrees of freedom and *P* value noted<br>*Give P values as exact values whenever suitable.*
☒	☐	For Bayesian analysis, information on the choice of priors and Markov chain Monte Carlo settings
☐	☒	For hierarchical and complex designs, identification of the appropriate level for tests and full reporting of outcomes
☐	☒	Estimates of effect sizes (e.g. Cohen's *d*, Pearson's *r*), indicating how they were calculated

Our web collection on statistics for biologists contains articles on many of the points above.

Software and code

Policy information about availability of computer code

Data collection	All the data used in this study were either collected by the authors in previous work or obtained from various publicly available sources as described in the Methods section. Restructuring of the data prior to the analyses was done in the R coding environment.
Data analysis	All the data analyses were performed in software R version 4.3.2. All the R code necessary to reproduce the results and figures of the present study were shared for review and are available via figshare at https://doi.org/10.6084/m9.figshare.25565169

For manuscripts utilizing custom algorithms or software that are central to the research but not yet described in published literature, software must be made available to editors and reviewers. We strongly encourage code deposition in a community repository (e.g. GitHub). See the Nature Portfolio guidelines for submitting code & software for further information.

Data

Policy information about availability of data

All manuscripts must include a data availability statement. This statement should provide the following information, where applicable:

- Accession codes, unique identifiers, or web links for publicly available datasets
- A description of any restrictions on data availability
- For clinical datasets or third party data, please ensure that the statement adheres to our policy

All data used in the analysis are available via figshare atAll data and code required to reproduce the results of this study were shared for review and will be available

via figshare at https://doi.org/10.6084/m9.figshare.25565169. These data result from the work of several people who applied for grants, sampled the tree plots and kept long-term plots running at great expenses. As such, it would be appreciated if data owners were consulted and invited for any publications using this dataset.

Research involving human participants, their data, or biological material

Policy information about studies with human participants or human data. See also policy information about sex, gender (identity/presentation), and sexual orientation and race, ethnicity and racism.

Reporting on sex and gender	This study does not involve human-related data, therefore no gender analysis were performed.
Reporting on race, ethnicity, or other socially relevant groupings	This study does not address human-related issues, therefore no social groupings were used.
Population characteristics	This study does not include any human-related data, therefore no population characteristics were described.
Recruitment	This study did not involve human participants, therefore no recruitment was done.
Ethics oversight	No specific ethic protocol was used as the present study does not involve any ethical issues and does not include any human-related data.

Note that full information on the approval of the study protocol must also be provided in the manuscript.

Field-specific reporting

Please select the one below that is the best fit for your research. If you are not sure, read the appropriate sections before making your selection.

☐ Life sciences ☐ Behavioural & social sciences ☒ Ecological, evolutionary & environmental sciences

For a reference copy of the document with all sections, see nature.com/documents/nr-reporting-summary-flat.pdf

Ecological, evolutionary & environmental sciences study design

All studies must disclose on these points even when the disclosure is negative.

Study description	This study applies a causal inference framework using extensive floristic and plant trait data to investigate how consistent and predictable are the causal effects of landscape-scale forest loss, landscape configuration and local degradation on the functional profiles of tree assemblages in human-modified tropical forests.
Research sample	We used abundance and trait data from 1,207 tree species across 271 tropical forest plots in six human-modified regions of the Amazonian and Atlantic forests in Brazil.
Sampling strategy	The present study did not involve sampling of novel observations. All the data were either previous collected by the authors or retrieved from online data repositories as described in the methods section.
Data collection	All the floristic data were previously collected by the authors following standard vegetation inventory designs, including the identification of all trees with diameter at breast height (DBH) > 10 cm in forest plots of varying sizes (see Extended Data Table 1). The trait data were either collected by the authors or retrieved from online data repositories, mainly the TRY database. The data on land-use types used to calculate landscape metrics were retrieved from the MapBiomas public repository.
Timing and spatial scale	This study includes data from six Neotropical forest regions, which were collected at different times, between 2000 and 2015. However, we did not perform any temporal analyses, as we relied on one single temporal snapshot in each region. The spatial scale of the analyses was within regions, which comprised 18 to 87 forest plots.
Data exclusions	Exclusion criteria was adopted based on the trait data coverage of total plot-level tree abundance. The selected forest plots are those with at least 50% of species-level trait data coverage and at least 80% of total trait coverage of community abundance, the latter including genus-level trait data.
Reproducibility	This study did not include an a priori experimental design, but all the steps of our data analyses and modeling approach are detailed in the Methods section and reproducible based on the data and code shared in a public repository.
Randomization	This study did not include an a priori experimental design, and therefore randomization was not necessary.
Blinding	This study did not include an a priori experimental design, and therefore blinding was not necessary.

Did the study involve field work? ☐ Yes ☒ No

Reporting for specific materials, systems and methods

We require information from authors about some types of materials, experimental systems and methods used in many studies. Here, indicate whether each material, system or method listed is relevant to your study. If you are not sure if a list item applies to your research, read the appropriate section before selecting a response.

Materials & experimental systems

n/a	Involved in the study
☒	☐ Antibodies
☒	☐ Eukaryotic cell lines
☒	☐ Palaeontology and archaeology
☒	☐ Animals and other organisms
☒	☐ Clinical data
☒	☐ Dual use research of concern
☒	☐ Plants

Methods

n/a	Involved in the study
☒	☐ ChIP-seq
☒	☐ Flow cytometry
☒	☐ MRI-based neuroimaging

Plants

Seed stocks

This study did not use seed stocks or involved the collection of any plant materials, only previously sampled data were used.

Novel plant genotypes

This study did not produce or used novel plant genotypes.

Authentication

No seed stocks or novel genotypes were generated or used in this study, therefore no authentication was needed.
